# Beyond Dialysis Adequacy: Transportation Time and Pain as Quality-of-Life Predictors in Polish Hemodialysis Patients—A Single-Center Study

**DOI:** 10.3390/jcm15041423

**Published:** 2026-02-11

**Authors:** Stanisław Rączewski, Weronika Caban, Natalia Lemiszewska, Mikołaj Kuncewicz, Magdalena Mosakowska, Ewa Kotwica-Strzałek, Stanisław Niemczyk

**Affiliations:** 1 Department of Internal Medicine, Nephrology and Dialysis, Military Institute of Medicine-National Research Institute, 04-141 Warsaw, Polandsniemczyk@wim.mil.pl (S.N.); 2 Faculty of Medicine, Medical University of Warsaw, 02-091 Warsaw, Poland; 3 Department of Descriptive and Clinical Anatomy, Medical University of Warsaw, 02-004 Warsaw, Poland

**Keywords:** chronic kidney disease, hemodialysis, pain, quality of life, commute burden

## Abstract

**Background:** Dialysis adequacy (Kt/V) remains an essential marker of hemodialysis quality; however, it does not fully capture patients’ overall well-being. Growing evidence underscores the need for a more holistic, patient-centered approach that integrates clinical efficiency with factors affecting daily functioning and quality of life (QoL). **Objectives**: This study aimed to identify the key determinants of health-related quality of life (HRQoL) among Polish patients undergoing hemodialysis. **Methods**: Seventy hemodialysis patients from a single center completed the KDQOL-36 questionnaire and provided demographic and clinical data. Statistical analyses included Pearson’s and Spearman’s correlations, as well as multiple linear regression, to determine predictors of HRQoL. **Results**: The mean (SD) KDQOL summary score was 60.9 (17.3). Pain (B = −15.9, *p* < 0.001) and the need for additional dialysis sessions (B = −10.2, *p* = 0.008) were the strongest independent predictors of poorer HRQoL, collectively accounting for 28.6% of variance. Longer dialysis-related transportation time (r = −0.238, *p* = 0.03) and longer hemodialysis vintage (r = −0.254, *p* = 0.03) were also significantly associated with lower HRQoL, while dialysis adequacy showed no significant effect. **Conclusions**: Pain, additional dialysis sessions, and longer dialysis-related transportation time are key, modifiable contributors to reduced HRQoL in Polish hemodialysis patients. These findings underscore the importance of a patient-centered approach that supplements clinical measures with interventions targeting comfort, education, and accessibility. Incorporating structured pain management and improved transport into routine nephrology practice can meaningfully improve patient QoL.

## 1. Introduction

Health-related quality of life (HRQoL) is a crucial outcome measure in hemodialysis (HD) patients, as it is associated with mortality, hospitalization, and overall patient well-being [1]. The Kidney Disease Quality of Life Short Form (KDQOL-36) is one of the most widely validated instruments for measuring QoL in dialysis populations, demonstrating reliability across multiple domains [2,3]. Analysis of patient responses yields scores in five domains: physical component summary, mental component summary, burden of kidney disease, symptoms and problems associated with kidney disease, and effects of kidney disease [2].

Previous studies have demonstrated an association between HRQoL and disease management [1,4]. Common factors associated with lower QoL among chronic kidney disease patients include older age, lower education, poverty, and social dysfunction. Additionally, pain and non-adherence to dietary restrictions have been recognized as significant barriers to patient well-being; however, their relative importance compared to biochemical adequacy remains unclear. Current trends in nephrology suggest that the need for supplemental dialysis should be minimized through robust educational efforts. The occurrence of extra sessions often results from both patient-related factors, such as non-adherence to dietary and fluid restrictions, and clinical management challenges within the dialysis process itself [5].

Kt/V is an index used to quantify dialysis adequacy, calculated by comparing pre- and post-dialysis blood urea nitrogen levels. For decades, Kt/V has been a central component of dialysis adequacy guidelines. Current KDOQI guidelines recommend a minimum single-pool Kt/V of approximately 1.2 per dialysis session for thrice-weekly hemodialysis to ensure adequate waste removal [6].

This study aimed to identify which clinical and organizational factors most strongly influence HRQoL among HD patients. The examined variables included sociodemographic characteristics, dialysis adequacy, dialysis vintage, duration of each dialysis session, and a summary of dialysis-related transportation time to and from the dialysis center, including waiting time for medical transport after dialysis sessions, number of hospitalizations, and perceived pain.

While quality of life has been studied globally, there is a lack of recent data focusing on the specific logistical and clinical challenges within the Polish healthcare system. By identifying the impact of transportation time and pain in a representative urban cohort, this study aims to provide actionable insights for improving patient-centered care at the institutional and regional levels.

## 2. Materials and Methods

A single-center, cross-sectional analytical study was conducted at the Dialysis Unit of the Department of Internal Medicine, Nephrology and Dialysotherapy at the Military Institute of Medicine—National Research Institute in Warsaw, Poland. The center serves the general civilian population as a tertiary care public hospital. The study group included adult patients undergoing maintenance hemodialysis, representative of the general population of hemodialysis patients in Poland

The study was approved by an institutional medical ethics committee at the Military Institute of Medicine—National Research Institute in Warsaw, Poland (decision number 34/25, 25 June 2025). All participants provided written informed consent to participate in the study. All patient personal details were anonymized and kept confidential throughout the analysis and reporting process.

The study cohort consisted of 70 hemodialysis patients who consented to participation and met the inclusion criteria, as follows: (1) age ≥ 18 years, (2) undergoing maintenance hemodialysis, and (3) clinical stability. Patients with acute illness, severe cognitive impairment, or inability to complete questionnaires were excluded. Participants completed the KDQOL-36 questionnaire and a demographic survey that included age, gender, education level, marital status, employment status, necessity of additional dialysis in last 6 months, and etiology of kidney disease. Additional clinical data were collected, including previous hospitalizations (within the past 6 months), dialysis frequency (in sessions per week), and commute time to and from the dialysis center, including waiting time for organized medical transportation. For the exploratory analysis of the study population, patients were stratified into two subgroups based on their health-related quality of life outcomes. The required criterion for this split was the mean KDQOL summary score calculated for the entire cohort (60.91 points). Patients achieving a score above this threshold were categorized into the ‘Higher QoL’ group, while those below were assigned to the ‘Lower QoL’ group.

In the KDQOL-36 questionnaire, each subscale score ranges from 0 to 100, with higher scores indicating better HRQoL. Thus, a higher score reflects fewer symptoms, lower perceived burden, or better physical and mental well-being, depending on the subscale. In contrast, lower scores indicate greater symptom severity, higher disease burden, or poorer functioning in the respective domains.

The questionnaire was administered either through face-to-face interviews or as self-administered questionnaires, depending on the patient’s preference. Participants were informed that they could decline to answer any question or withdraw from the study at any time. All responses were collected during dialysis sessions at the facility. All patient data were anonymized immediately after collection and assigned identification numbers for data analysis.

Descriptive statistics, including means, standard deviations, and frequencies, were calculated for all variables. Before statistical analysis, the normality of KDQOL values was confirmed using the Shapiro–Wilk test. For bivariate associations, Pearson’s and Spearman’s correlation coefficients were used for normally distributed continuous variables, and Spearman’s rho for non-parametric data. Independent samples t-tests compared group means (additional dialysis vs. no additional dialysis; pain present vs. absent), while chi-square tests evaluated associations between categorical variables. To identify independent predictors of KDQOL scores, multiple linear regression analysis was performed, entering all significant univariate variables. Statistical significance was set at *p* < 0.05. The strength r—Pearson correlation coefficient. was interpreted as follows: |r| < 0.3—weak; 0.3 ≤ |r| < 0.5—moderate; |r| ≥ 0.5—strong relationship. Positive values indicate a direct correlation, while negative values indicate an inverse relationship. All analyses were conducted using IBM SPSS version 29 Statistical software.

## 3. Results

### 3.1. Sociodemographic Characteristics

Sociodemographic characteristics are summarized in Table 1. The total number of patients included in the study cohort was 70. The patients’ ages ranged from 36 to 90 years, with a mean of 68.24 years (SD = 11.08 years). The majority of participants were male, at 61.4%. A total of 28 individuals (40.0%) were married. Most participants were not working, with 58 (82.9%) mostly retired and 46 (65.7%) retired. The predominant level of education completed was secondary, with 44 (62.9%) individuals. To further explore the data, the study population was divided into two subgroups based on the mean KDQOL summary score (60.91 points). The first group consisted of 34 patients (48.6%) with scores above the mean (KDQOL > Mean), representing better health-related quality of life, while the second group included 36 patients (51.4%) with scores below the mean (KDQOL < Mean). No significant differences in age or gender distribution were observed between the groups, although a trend toward lower quality of life was noted in older patients.

### 3.2. Clinical Characteristics

Clinical characteristics of the study population are summarized in Table 2. The most common cause of kidney disease was chronic kidney disease associated with diabetes, being the leading cause among 19 (27.1%) participants. All patients underwent scheduled hemodialysis sessions three times per week, with the mean (SD) dialysis duration of 3.48 (0.40) hours. The mean (SD) single-pool Kt/V obtained in our study group was 1.17 (0.24), which is below the minimal value of 1.2 and target dose of 1.4 recommended by the KDIGO guidelines. The mean (SD) of HD vintage was 45.36 (40.60) months. During the previous 6 months, 38.6% of patients required at least one additional dialysis session, and 58.6% were hospitalized, with a mean (SD) hospitalization duration of 11.14 (18.74) days.

### 3.3. Baseline QoL Characteristics

Quality of Life Characteristics, based on the five KDQOL-36 subscales and two summary scores, are shown in Table 3. The highest mean score was observed on the Symptoms and Problems subscale (74.97), indicating that, on average, patients reported relatively few symptoms or concerns. While the Burden of Kidney Disease subscale had the lowest mean score (44.1), indicating a higher perceived burden of the disease among patients, it also showed the broadest score range (SD of 30.43).

### 3.4. Correlation Between Sociodemographic and Clinical Data

#### 3.4.1. Sociodemographic Variables and Clinical Parameters

Sociodemographic variables, such as age (B = −0.06, *p* = 0.72), sex (B = −1.32, *p* = 0.73), education (B = 3.57, *p* = 0.26), and marital status (B = 1.39, *p* = 0.39), did not show significant associations with the KDQOL scores.

Experiencing pain was negatively correlated with all KDQOL subscales, indicating a strong negative association with quality of life across all domains.

Hemodialysis vintage was negatively correlated with SF-12 Physical component scores (*p* = 0.03), meaning that a longer duration of HD treatment was associated with lower physical health scores. No significant correlations were observed between hemodialysis vintage and KDQOL subscale scores. Similarly, Kt/V values showed no significant correlation with any of the assessed quality-of-life measures. The values are presented in Table 4.

#### 3.4.2. Need for Additional Dialysis Sessions

Patients requiring additional dialysis had significantly lower KDQOL scores in all KDQOL subscales (*p* < 0.05), except for the SF-12 mental health composite. A comparison of mean KDQOL subscale scores is presented in Table 5. The results indicate poorer physical health and a greater disease burden among patients requiring additional dialysis. The lack of a significant difference found in the SF-12 mental health composite (t = 0.789, *p* = 0.22) suggests that the groups have comparable mental health statuses.

#### 3.4.3. Total Dialysis Transportation Burden, Dialysis Duration

Comparison between commute and dialysis times is shown in Table 6. Longer dialysis-related transportation time (including waiting for organized medical transport) to the hemodialysis site was significantly associated with lower scores on the effects of kidney disease subscale (r = −0.258; *p* = 0.047) and the general KDQOL score (r = −0.238; *p* = 0.03). Dialysis session duration showed no significant association with any of the subscales.

#### 3.4.4. Regression Analysis

Prior to multivariable modeling, univariate linear regression analyses were performed to examine the crude associations between each candidate predictor and KDQOL total score. Variables considered included age, sex, educational level, marital status. Variables showing a univariate association with KDQOL at *p* < 0.20, as well as variables considered clinically relevant based on prior literature, were retained for multivariable analysis.

A multiple linear regression model was then constructed to identify independent predictors of KDQOL while adjusting for potential confounders. KDQOL total score was treated as the dependent variable, and age, sex, educational level, marital status, additional dialysis sessions, and pain experience were entered simultaneously as independent variables. These covariates were selected a priori due to their plausible associations with quality of life in patients undergoing dialysis.

The overall model was statistically significant (F(6, 63) = 5.604, *p* < 0.001), indicating that the included predictors collectively explained a significant proportion of the variance in KDQOL scores. The adjusted coefficient of determination (adjusted R^2^ = 0.286) suggested that approximately 28.6% of the variability in KDQOL was explained by the model, which is considered acceptable in observational studies of quality-of-life outcomes. Model assumptions, including linearity, homoscedasticity, normality of residuals, and absence of multicollinearity, were assessed and found to be acceptable.

After adjustment, pain experience (B = −15.93, *p* < 0.001) and additional dialysis sessions (B = −10.18, *p* = 0.008) were independently associated with lower KDQOL scores. Specifically, patients reporting pain had, on average, a 15.9-point lower KDQOL score compared with those without pain, while patients requiring additional dialysis sessions had a 10.2-point lower KDQOL score. Other covariates were not significantly associated with KDQOL in the multivariable model.

## 4. Discussion

Our patients’ demographic and treatment characteristics align with those observed in international HD populations assessed with the KDQOL-36 (Table 1) [4], except for markedly higher summary commute time. Our cohort demonstrates poor physical HRQoL and moderate kidney-disease-specific quality of life (Table 3), consistent with reports from the United States [3] and Polish populations [7]. Notably, the Symptoms and Problems subscale showed the highest scores. In contrast, the Burden of Kidney Disease subscale showed the lowest scores, indicating that patients experience a significant disease burden despite relatively few reported symptoms, which is consistent with previous studies.

Pain represents one of the most prevalent yet under-treated symptoms among patients with chronic kidney disease, with systematic review evidence demonstrating that approximately 60% of patients undergoing hemodialysis experience pain, significantly exceeding the 20% prevalence in the general population [5,8]. Our finding, that pain emerged as the strongest independent predictor of reduced health-related quality of life among all subscales (B = −15.9, *p* < 0.001) and explained substantial variance in HRQoL, aligns with this evidence and highlights the profound impact of pain on patient well-being. Patients undergoing hemodialysis face multiple pain sources, including procedure-related discomfort from repeated vascular access punctures, muscle cramps during or after dialysis sessions, and pain from prolonged sitting during treatments. Beyond dialysis-specific pain, many patients experience chronic musculoskeletal pain- the most common pain type in hemodialysis populations, affecting approximately 45% of patients. Musculoskeletal pain is often associated with mineral and bone disorders that accompany chronic kidney disease, which disrupt calcium and phosphorus homeostasis and increase the risk of fractures. Neuropathic pain, present in approximately 10% of patients, includes diabetic peripheral neuropathy among patients with chronic kidney disease associated with diabetes (27.1% in our cohort) and uremic neuropathy. These diverse pain sources create a complex situation where conventional analgesic approaches often fail to alleviate the pain adequately. Despite this high burden, pain remains systematically under-treated in dialysis populations, mainly due to limited awareness among healthcare providers and concerns about analgesic safety in patients with impaired kidney function [9,10]. Our results, showing that 60% of patients experienced pain and that pain was negatively correlated with all KDQOL-36 subscales, underscore the urgent need for comprehensive pain assessment and management strategies integrated into routine dialysis care. Evidence-based interventions should address multiple pain mechanisms, including optimizing vascular access techniques to minimize puncture-related pain, implementing intradialytic positioning protocols to reduce musculoskeletal discomfort, ensuring adequate management of bone mineral metabolism, and providing appropriate pharmacological treatment tailored to individual renal function. For neuropathic pain, which systematic review evidence identified as significantly associated with diabetes, hypertension, and higher BMI [8], specific agents such as gabapentin may be indicated, though dosing requires careful adjustment. Non-pharmacological approaches, including physical therapy, heat application for muscle cramps, and cognitive-behavioral interventions, also warrant consideration [8,10,11]. Given that pain was the single strongest predictor of quality of life in our cohort. Healthcare systems should prioritize systematic pain screening using validated tools, multidisciplinary pain management protocols, and staff education on pain recognition and treatment. Such comprehensive pain management strategies represent a critical yet underutilized opportunity to improve outcomes for hemodialysis patients significantly.

The negative correlation between longer duration of HD treatment and lower physical health scores may result from aging and the progression of underlying disease, leading to a decline in general condition commonly observed among elderly patients undergoing HD. However, this is not associated with increased mortality in this group [6,12].

The mere duration of the HD session was not associated with quality of life, which may be attributed to very similar dialysis times for most participants. Moreover, longer dialysis sessions have been associated with better survival and intermediate outcomes. Still, they may negatively impact social well-being, suggesting a complex relationship between the length of HD sessions and quality of life [13,14].

Dialysis adequacy in our cohort generally meets the minimal targets, with a mean single-pool Kt/V of 1.17. However, other cohorts achieve higher average Kt/V values, ranging from 1.4 to 1.5 [6,15]. We found no effect of dialysis adequacy on quality of life, and the absence of correlation underscores the limitations of depending exclusively on biochemical markers. This underscores the need to identify additional significant variables to enable more personalized care. An important observation in this study was that patients requiring additional dialysis sessions demonstrated significantly lower quality of life compared with those treated exclusively on a standard thrice-weekly schedule. This reduction was reflected in both the overall KDQOL summary score and in nearly all subscales. Although this may be partially explained by factors such as a higher uremic burden, greater comorbidity load, or inadequate clearance, it is most likely caused by non-adherence to fluid intake or dietary recommendations, leading to fluid overload and a subsequent need for extra dialysis. Implementing a structured educational process could address these gaps, reducing the need for treatment intensification and improving overall patient outcomes. Given that non-compliance with dietary and fluid guidelines is common among HD patients, the necessity of additional dialysis sessions should alert clinicians’ attention [4,6,16]. Non-compliance results in a lower quality of life, a higher risk of hospitalizations, adverse outcomes, and even death, making it especially important to personalize care and recommendations and motivate patients accordingly. Additionally, the negative association between additional dialysis sessions and KDQOL suggests that either the factors leading to additional dialysis or increased dialysis burden may contribute to lower patient well-being. Interventions in this area could significantly improve adherence to restrictions, but they should include a cognitive or cognitive/behavioral component [1,4,17].

Dietetic support plays a pivotal role in enhancing adherence to dietary and fluid intake restrictions among HD patients. Non-adherence to these restrictions is prevalent, with studies indicating rates as high as 60%, which can lead to adverse health outcomes [18]. Engagement with dietetics professionals has been shown to address this issue effectively. For instance, structured nutrition education programs have demonstrated significant improvements in patients’ adherence to dietary and fluid restrictions, as evidenced by reductions in interdialytic weight gain and improved blood pressure control [19].

Recent qualitative research from Spain further underscores the importance of dietitian involvement. Patients report that consistent, culturally sensitive guidance from dietitians empowers them to manage their dietary restrictions more effectively, resulting in improved health outcomes. Furthermore, caregivers recognize the support of renal dietitians as a crucial facilitator in assisting patients to adhere to dietary recommendations [11].

Incorporating dietetics professionals into the multidisciplinary care team is crucial for developing individualized dietary plans that account for patients’ cultural and personal preferences. Such tailored interventions improve adherence and patients’ quality of life by reducing the burden of dietary restrictions. The systematic review also suggests including cognitive or cognitive-behavioral components in such interventions [17].

Therefore, healthcare systems should prioritize integrating dietetic services into routine HD care to optimize patient outcomes. In the context of the Polish healthcare system, this underscores the need to integrate dietitian services into primary care for patients on hemodialysis. Such integration would enhance adherence to dietary and fluid intake recommendations, thereby improving patient outcomes.

### 4.1. Clinical Dialysis-Related Transportation Time

A longer combined commute and wait time to the dialysis site was significantly associated with lower HRQoL. This result is consistent with extensive international studies, which found that longer travel times were associated with a higher adjusted relative risk (RR) of death and worse HRQoL in HD patients across multiple subscales, even after adjusting for other factors [20,21]. Our findings regarding transportation time are particularly relevant in the context of the Polish healthcare infrastructure, suggesting that logistical improvements could be as impactful as clinical interventions in enhancing patient well-being. The negative impact of a long commute can be attributed to several causes. Foremost, transportation itself is time-consuming, effectively adding on to the “dialysis day” and reducing time for rest, family, or work [22]. It may also limit social activities and cause additional fatigue, pain (if transportation is uncomfortable), and stress. At least some patients in this HD station rely on ambulance transportation to our dialysis station, which may be associated with longer waiting and commute times due to inefficient transport, for example, waiting for other patients to complete dialysis. Consequently, even the patients who live close to the dialysis center, if they have reduced mobility, have to rely on group, time-consuming, and often delayed transport.

In various countries, alternatives to ambulance transport for HD patients have been implemented. For example, in multiple municipalities in the United States of America, patients may use private cars, taxis, or wheelchair-accessible vehicles to travel between their residences and healthcare facilities. Permission for such transport is granted based on medical assessments, and patients can receive reimbursement for these journeys [23].

The problem could also be improved by increasing the availability of home HD. Currently, in Denmark, home HD accounts for 7% of all dialysis treatments. It is mainly delivered using dialysis machines installed at no cost in patients’ homes. Patients typically begin as limited-care in-center HD patients and transition to home hemodialysis after 3–6 months of training. Some centers in Denmark have adopted a “Home HD First” policy, resulting in higher home hemodialysis adoption rates (up to 14% in these centers). The policy emphasizes autonomy, improved outcomes, and cost-effectiveness. Additionally, Denmark has one of the highest rates of home dialysis in Europe, with 28% of patients treated at home, 21% with peritoneal dialysis, and 7% with home hemodialysis [24].

### 4.2. Limitations

There are several significant limitations to our findings. Most data were self-reported, sometimes via a single, non-validated question. Secondly, there could be substantial nonresponse bias. Non-responders were usually older, possibly feeling worse during HD, and therefore not keen to participate in the survey. Thirdly, this study was conducted at a single center, limiting the generalizability of the findings. Furthermore, the cross-sectional, single-time-point design of the study precludes the assessment of changes over time and does not allow for causal inferences regarding the identified predictors. Although the mean dialysis adequacy (Kt/V) in our study population did not meet the target threshold recommended by KDIGO guidelines, we did not observe a significant impact of this parameter on the reported quality of life outcomes or other primary findings. Our study did not include a formal nutritional assessment or sarcopenia evaluation. Furthermore, our study did not analyze the specific reasons for supplementary dialysis sessions or the extent of patient education, which are critical for optimizing the treatment process and avoiding unnecessary dialysis intensification. Validation in a multi-center setting is warranted.

## 5. Conclusions

These results underscore the need for a more personalized, holistic model of dialysis care that balances clinical efficacy with patient comfort, access to services, and quality of life. In the context of Polish nephrology, particular attention should be paid to improving pain control, facilitating dietary adherence, and reducing logistical barriers to treatment. Further studies are needed regarding how individually tailored education programs, standardized pain management plans, and transportation support can be effectively integrated into routine, patient-centered dialysis care. Future research should focus on longitudinal assessments to track QoL dynamics and intervention-based studies evaluating the impact of dedicated transportation services and structured nutritional support. Furthermore, multicenter cohorts are warranted to confirm these findings on a national scale.

## Figures and Tables

**Table 1:** Sociodemographic characteristics of the study population.

Variable	Female	Male	KDQOL > Mean	KDQOL < Mean	All (%)
**Age group (years)**	**27 (38.6)**	**43 (61.4)**	**34 (48.6)**	**36 (51.4)**	70
<65	6 (22.2)	13 (30.0)	11 (32.4)	8 (22.2)	19 (27.1)
≥65	21 (77.8)	30 (70.0)	23 (67.6)	28 (77.8)	59 (84.3)
**Marital Status**					
Married	6 (22.2)	22 (51.2)	14 (41.1)	14 (38.8)	28 (40.0)
Single	4 (14.8)	7 (16.3)	7 (20.6)	4 (11.1)	11 (15.7)
Widowed	13 (48.1)	8 (18.6)	10 (29.4)	11 (30.5)	21 (30.0)
Divorced	4 (14.8)	6 (13.9)	3 (8.8)	7 (19.4)	10 (14.3)
**Occupation**					
Employed	6 (22.2)	6 (13.9)	5 (14.7)	7 (19.4)	12 (17.1)
Unemployed	1 (3.7)	2 (4.7)	2 (5.9)	1 (2.7)	3 (4.3)
Retired	19 (70.4)	27 (62.8)	22 (64.7)	24 (66.7)	46 (65.7)
Medical pension	1 (3.7)	8 (18.6)	5 (14.7)	4 (11.1)	9 (12.9)
**Educational status**					
Primary	2 (7.4)	3 (7.0)	2 (5.9)	3 (8.3)	5 (7.1)
Secondary	17 (63.0)	27 (62.8)	21 (61.8)	23 (63.9)	44 (62.9)
Tertiary	8 (29.6)	13 (30.2)	11 (32.3)	10 (27.8)	21 (30.0)

Showing number of patients (percentage).

**Table 2:** Clinical characteristics of the study population.

Variable	Frequency (%)	Mean (SD)
**Dialysis per week**		3.00 (0)
**Vintage (months of HD)**		45.36 (40.60)
**Dialysis duration (hours)**		3.48 (0.40)
**Kt/V**		1.17 (0.24)
**Commute time (hours)** ^1^		4.89 (2.77)
**Additional dialyses**		
Yes	27 (38.6)	
No	43 (61.4)	
**Hospitalization (last 6 months)**		
Yes	41 (58.6)	11.14 (18.74) days
No	29 (41.4)	
**Experienced pain**		
Yes	42 (60.0)	
No	28 (40.0)	
**Etiology of kidney disease**		
Chronic kidney disease associated with diabetes	19 (27.1)	
Hypertensive nephropathy	2 (2.9)	
Obstructive uropathy	7 (10.0)	
Polycystic kidney disease	7 (10.0)	
Unclear	18 (25.7)	
Other	17 (24.3)	

^1^ Total commute time-including the wait for organized medical transport; SD, Standard Deviation; HD, Hemodialysis.

**Table 3:** Baseline characteristics, *n* = 70.

Component	Mean	SD
Summary KDQOL score	60.91	17.33
Symptoms and problems	74.97	16.47
Effects of the kidney disease	65.63	19.86
Burden of the kidney disease	44.10	30.43
SF-12 Physical health composite	35.28	11.47
SF-12 Mental health composite	49.03	11.58

Scores range from 0 to 100, where higher values indicate better health-related quality of life.

**Table 4:** Correlation between quality of life and dialysis parameters.

Variable	Vintage of HD	Kt/V Value	Pain
KDQOL	−0.187	−0.039	−0.524 *
Symptoms and problems	−0.186	−0.001	−0.493 *
Effects of the kidney disease	−0.07	−0.014	−0.246 *
Burden of the kidney disease	−0.116	−0.034	−0.305 *
SF-12 Physical	−0.254 *	−0.014	−0.340 *
SF-12 Mental	0.021	−0.07	−0.729 *

r values from Pearson’s and Spearman’s correlations; statistically significant correlations (*p* < 0.05) are shown in bold and marked with *.

**Table 5:** Comparison of KDQOL mean scores based on the need for additional dialysis.

Additional Dialyses	*n*	KDQOL	Symptoms and Problems	Effects of the Kidney Disease	Burden of the Kidney Disease	SF-12 Physical	SF-12 Mental
Required	27	53.73 *	70.66 *	56.83 *	34.49 *	27.58 *	47.70
Not required	43	65.42 *	77.66 *	71.15 *	50.15 *	35.23 *	49.95

Statistically significant correlations (*p* < 0.05) are shown in bold and marked with *; means compared using Student’s *t* test; Scores range from 0 to 100, where higher values indicate better health-related quality of life.

**Table 6:** Correlation of HRQoL with commute and dialysis durations.

Variable	Mean (SD)	KDQOL	Symptoms and Problems	Effects of the Kidney Disease	Burden of the Kidney Disease	SF-12 Physical	SF-12 Mental
Commute time ^1^	4.89 (2.77)	−0.258 *	−0.139	−0.238 *	−0.150	−0.205	−0.110
Dialysis time	3.48 (0.40)	−0.160	−0.188	−0.210	−0.058	−0.131	0.021

^1^ Total commute time, including the wait for organized medical transport; SD, Standard Deviation r values from Pearson’s and Spearman’s correlations; statistically significant correlations (*p* < 0.05) are shown in bold and marked with *.

## Data Availability

The data presented in this study are available on request from the corresponding author. The data are not publicly available due to privacy and ethical restrictions, as they contain information that could compromise the anonymity of the research participants.

## Abbreviations

The following abbreviations are used in this manuscript:

BMI	Body Mass Index
CKD	Chronic Kidney Disease
HD	Hemodialysis
HRQoL	Health-Related Quality of Life
KDOQI	Kidney Disease Outcomes Quality Initiative
KDQOL	Kidney Disease Quality of Life
Kt/V	Dialysis Adequacy Index
QoL	Quality of Life
RR	Relative Risk
SD	Standard Deviation
SF-12	Short Form 12 Health Survey
SPSS	Statistical Package for the Social Sciences
